# Variation in breast cancer grading in 1,636 resections assessed using control charts and in silico kappa

**DOI:** 10.1371/journal.pone.0242656

**Published:** 2020-12-28

**Authors:** Jinesa Moodley, Phillip Williams, Gabriela Gohla, Pierre Major, Michael Bonert

**Affiliations:** 1 Pathology and Molecular Medicine, McMaster University, Hamilton, Ontario, Canada; 2 Laboratory Medicine and Pathobiology, University of Toronto, Toronto, Ontario, Canada; 3 Medical Oncology, McMaster University, Hamilton, Ontario, Canada; CNR, ITALY

## Abstract

**Objective:**

Assess interpretative variation in Nottingham grading using control charts (CCs) and in silico kappa (ISK).

**Methods:**

In house invasive breast cancer cases (2011–2019) at two institutions with a synoptic report were extracted. Pathologist interpretative rates (PIRs) were calculated and normed for Nottingham grade (G) and its components (tubular score (TS), nuclear score (NS), mitotic score (MS)) for pathologists interpreting >35 cases. ISKs were calculated using the ordered mutually exclusive category assumption (OMECA) and maximal categorical overlap assumption (MCOA).

**Results:**

The study period included 1,994 resections. Ten pathologists each assessed 38–441 cases and together saw 1,636; these were further analyzed. The PIR medians (normed ranges) were: G1:24%(18–27%), G2:53%(43–56%) and G3:26%(19–33%). The MCOA ISK and the number of statistical outliers (p< 0.05/p< 0.001) to the group median interpretive rate (GMIR) for the ten pathologists was G1: 0.82(2/0 of 10), G2: 0.76(1/1), G3: 0.71(3/1), TS1: 0.79(1/0), TS2: 0.63(5/1), TS3: 0.66(5/1), NS1: 0.37(5/4), NS2: 0.60(4/3), NS3: 0.59(4/4), MS1: 0.78(3/1), MS2: 0.78(3/1), MS3: 0.77(2/0). The OMECA ISK was 0.62, 0.49, 0.69 and 0.71 for TS, NS, MS and G.

**Conclusions:**

The nuclear score has the most outliers. NS1 appears to be inconsistently used. ISK mirrors trends in conventional kappa studies. CCs and ISK allow insight into interpretive variation and may be essential for the next generation in quality.

## Introduction

Grading of breast carcinoma has been developed and modified over more than sixty years. Major contributors to the grading system includes Patey and Scarff and Bloom and Richardson [1]. Patey and Scarff proposed tubule formation, variation in nuclear size and mitotic figures as variables important in grading in 1928 [1]. Later in 1957 Bloom and Richardson re-examined Greenhough’s method to include three factors; degree of tubular arrangement, nuclear pleomorphism and frequency of mitotic figures [2]. Elston and Ellis later modified the Scarff Bloom Richardson grading system in 1991, which is now referred to as the Nottingham Grading System [3]. The Nottingham Grading system has been recommended by the World Health Organization [4] and is the most widely used grading system for breast carcinoma [2]. This grading system has been shown to significantly correlate with long term prognosis, as patients with a grade 1 tumor have an 85% chance of surviving 10 years after diagnosis, whereas those with grade 3 tumors have a 45% chance [2].

Interobserver variability in grading breast carcinoma has been studied extensively [1,2,3,5–11]. Findings have varied considerably in the reproducibility of the Nottingham grade and its components. Frierson *et al* and Robbins *et al*, describe the Nottingham grade as having an excellent reproducibility when used by experienced pathologists [2]. Frierson *et al* showed pairwise kappa values for agreement ranging from moderate to substantial (0.43–0.74) for overall (histologic) grade [5]. With regards to the Nottingham grade components, substantial agreement was shown for tubular score (k = 0.64), moderate for mitotic score (k = 0.52) and near moderate agreement for nuclearscore (k = 0.40) [5]. Meyer *et al* describe the Nottingham grade as being modestly reproducible [4]. They specifically showed that agreement of nuclear score was less than good by kappa statistic (k = 0.38) [1]. With regards to the overall grade, disagreements of one step (grade 1 versus grade 2, grade 2 versus grade 3) are common, discrepancies greater than one rarely occur [1]. Zhang *et al* looked at inter-observer variability among four general pathologists [4]. They showed a fairly reproducible kappa of 0.34 in the Nottingham score [4]. The best agreement was in tubular score (k = 0.46), intermediate for nuclear score (k = 0.42) and poorest for mitotic score (k = 0.28) [4]. Of the randomly selected cases among the four general pathologists, complete agreement on grade was only achieved in 29% (29/100) [4]. Sixty-one cases were graded as either of two adjacent grades and 10 from grades 1–3 [4]. Atanda et al showed moderate agreement in twenty-four cases on which inter-rater agreement was tested (k = 0.53) [10]. Fair agreement was shown for both mitotic score and nuclear score (k = 0.25 and 0.34, respectively) [10]. Tubular score showed moderate agreement (k = 0.57) [10]. Overall pairwise kappa agreement from Atanda *et al* ranged from fair to good (k = 0.31–0.63) [10]. Boiesen *et al* included slides from 93 invasive breast cancers to seven pathology departments in the southern healthcare region of Sweden [11]. Thirty-one percent of the cases obtained the same histologic grade for all departments [11]. Moderate reproducibility was obtained for overall grade, with a mean kappa of 0.54 [11]. In keeping with other studies, tubular score was best (k = 0.61), versus nuclear score (k = 0.44), and mitotic score (k = 0.46) [11]. In summary, throughout the literature, tubular formation has been shown to have the best agreement, as opposed to nuclear pleomorphism and mitotic activity.

Statistical process control (SPC), also known as Next Generation Quality (NGQ), is a method used to manage processes over time, using periodic measurements and feedback to achieve a target range and/or maintain a steady performance. SPC/NGQ is used for process monitoring (quality assurance) and process improvement (quality improvement). Quality improvement in SPC/NGQ is facilitated by plotting control parameter trajectories, such that undesirable trajectories can be understood and counteracted. SPC/NGQ is widely applied in manufacturing to achieve consistently high levels of quality and has a long history [12]. It has been used in health care [13]; however, it is not frequently applied in anatomical pathology.

This study examines interpretative rate variation (which can be used to infer inter-rater variation) in the histologic grading of invasive breast cancer. It makes use of control charts, a tool of statistical process control (SPC)/Next Generation Quality [14,15]. To bridge the world of (conventional) kappa studies with observational data, a novel in silico kappa is used.

## Materials and methods

Ethics approval (Hamilton Integrated Research Ethics Board (HiREB) # 4396) was obtained to retrieve all in house surgical pathology accessioned 2011–2019 at two affiliated teaching hospitals, such that all in house breast pathology cases could be retrieved. Patient consent was not required by the ethics board, due to the study design. The study was done in accordance with national ethics guidelines and relevant regulations. As the study had no research subjects, the requirement for informed consent from subjects is not applicable.

The data extraction and analysis included several steps. It follows a process established from other projects done [16]. An overview of this process is:

1. extraction of all in house pathology reports for the time period (accessioned Jan 1, 2011-Dec 31, 2019) from the laboratory information system
2. complete irreversible removal of all patient identifiers using custom computer code (CCC) written in python (https://python.org)
3. retrieval of all cases with an invasive breast cancer (College of American Pathologists) synoptic report and reconstruction of the categorical data (using CCC written in python)
4. anonymiztion of healthcare providers (using CCC written in python)
5. tabulation of cases in LibreOffice Calc and with CCC written in python
6. construction of funnel plots and control charts with CCC written in GNU/Octave (https://www.gnu.org/software/octave/)
7. calculation of in silico kappa with CCC written in GNU/Octave

Pathologist interpretative rates (PIRs) for the Nottingham grade (G) and its components (tubular score (TS), nuclear score (NS), mitotic score (MS)) were calculated for pathologists interpreting >35 cases.

### Funnel plots and control charts

Consistency was assessed using funnel plots (FPs) centred on the group median interpretative rate (GMIR) with control limits that defined the 95% and 99.9% confidence interval (CI) in relation to the GMIR.

PIRs were normed using (1) maximal cases read, and (2) the standard deviations to the GMIR for each pathologist (details of this procedure are in Appendix A). Normalization was done to get a common scale for comparisons.

The normalization done masks the case volume and thereby ensures greater anonymity for the providers. Also, the normalized (control) charts are easier to read than funnel plots as the providers are separated; data points do not overlap and may be less confusing.

The normed PIRs were plotted on control charts (CCs) with the GMIR and control lines that defined the 95% CI, 99.9% CI, 100*(1-1e-6)% CI, and 100*(1-1e-12)% CI.

The normalized plots (control charts) are essentially a PIR comparison based on the number of standard deviations (SDs) to the GMIR; however, the SDs are converted to (PIR) percentages, as percentages are easier to visualize than SDs. In most cases, the normalization shrinks the interpretative rate range. Normalization can also make PIRs of zero into non-zero PIRs (see Supplemental Material for details).

### In silico kappa

In silico kappas (ISKs) were calculated using the PIRs. Kappa scores for the individual scores within a category (e.g. TS1) were calculated with the maximal categorical overlap assumption (MCOA). The kappa scores for the complete interpretative category (e.g. tubular score) were done with the ordered mutually exclusive category assumption (OMECA). Details of the calculation are found in Appendix B. Conceptually, MCOA is similar to the kappa maximum described by Sim and Wright [17].

ISK using the maximal categorical overlap assumption (MCOA) is a measurement of the PIR differences in the group of pathologists; it assumes that pathologists with the same interpretative rates agree on all their interpretations in the (in silico) simulation.

ISK using the ordered mutually exclusive category assumption (OMECA) presumes that only adjacent (mutually exclusive) categories are considerations in the interpretative process. For example, if a pathologist is considering ‘nuclear score 1’, ‘nuclear score 2’ is a possibility but *not* ‘nuclear score 3’. ‘Nuclear score 1’ is adjacent to ‘nuclear score 2’; it is *not* adjacent to ‘nuclear score 3’. ‘Mutually exclusive’ refers to the fact that one category applies for a given case. A case cannot be simultaneously be ‘nuclear score 1’ and ‘nuclear score 2’; it is either one or the other. As the categories are ordered and mutually exclusive, the pathologist interpretative overlaps can be constructed (see Supplemental Materials for visual example). The amount of overlap determines the (OMECA) ISK.

## Results

The study period included 1,994 resections. 1,877 cases of the 1,994 had a grade and 409 were G1, 972 were G2 and 496 were G3. There were 117 cases that did not have a grade; these were analyzed by T stage: 39 pT0, 2 pT1a, 1 pT1c, 37 pT1mi, 7 pT2, 3 pT3, 24 pTis and 4 pTX.

PIRs for TS, NS, MS and G were calculated using (specific to each pathologist) the denominators TS1+TS2+TS3, NS1+NS2+NS3, MS1+MS2+MS3, and G1+G2+G3 respectively.

Ten pathologists interpreted 38–441 cases and together assessed 1,636; this is the number assessed in the further analysis The smallest G1+G2+G3 of the 10 pathologists examined in greater detail was 31.

The median PIRs, normed PIR ranges, number of statistical outliers (p< 0.05/p< 0.001) to the GMIR for the ten pathologists and ISK are summarized in Table 1.

**Table 1:** Summary of the findings.

Parameter	Med. PIR (Norm. Range)	Outliers p<0.05	Outliers p<0.001	In silico Kappa (95% CI)	Assumption
Tubular Score (TS)	NA	NA	NA	0.62 (0.51–0.67)	OMECA
TS1	9% (6–12)	1 of 10	0 of 10	0.79 (0.59–0.83)	MCOA
TS2	23% (17–32)	5 of 10	1 of 10	0.63 (0.52–0.72)	MCOA
TS3	66% (57–73)	5 of 10	1 of 10	0.66 (0.55–0.73)	MCOA
Nuclear Score (NS)	NA	NA	NA	0.49 (0.41–0.54)	OMECA
NS1	5% (0–11)	5 of 10	4 of 10	0.37 (0.24–0.45)	MCOA
NS2	57% (39–80)	4 of 10	3 of 10	0.6 (0.51–0.65)	MCOA
NS3	37% (20–59)	4 of 10	4 of 10	0.59 (0.49–0.62)	MCOA
Mitotic Score (MS)	NA	NA	NA	0.69 (0.53–0.75)	OMECA
MS1	57% (54–69)	3 of 10	1 of 10	0.78 (0.66–0.83)	MCOA
MS2	21% (14–27)	3 of 10	1 of 10	0.78 (0.64–0.81)	MCOA
MS3	20% (15–23)	2 of 10	0 of 10	0.77 (0.62–0.83)	MCOA
Nottingham Grade (NG)	NA	NA	NA	0.71 (0.57–0.76)	OMECA
NG1	24% (18–27)	2 of 10	0 of 10	0.82 (0.68–0.86)	MCOA
NG2	53% (43–56)	1 of 10	1 of 10	0.76 (0.65–0.82)	MCOA
NG3	26% (19–33)	3 of 10	1 of 10	0.71 (0.57–0.77)	MCOA

‘Med. PIR’ is median pathologist interpretative rate. ‘Norm. Range’ is the normalized interpretative range (see Appendix A). ‘MCOA’ is maximal diagnostic overlap assumption. ‘OMECA’ is ordered mutually exclusive category assumption. The median PIR sum nearly to 100%; it should not be surprise that they are not exactly 100%.

**Fig 1:**
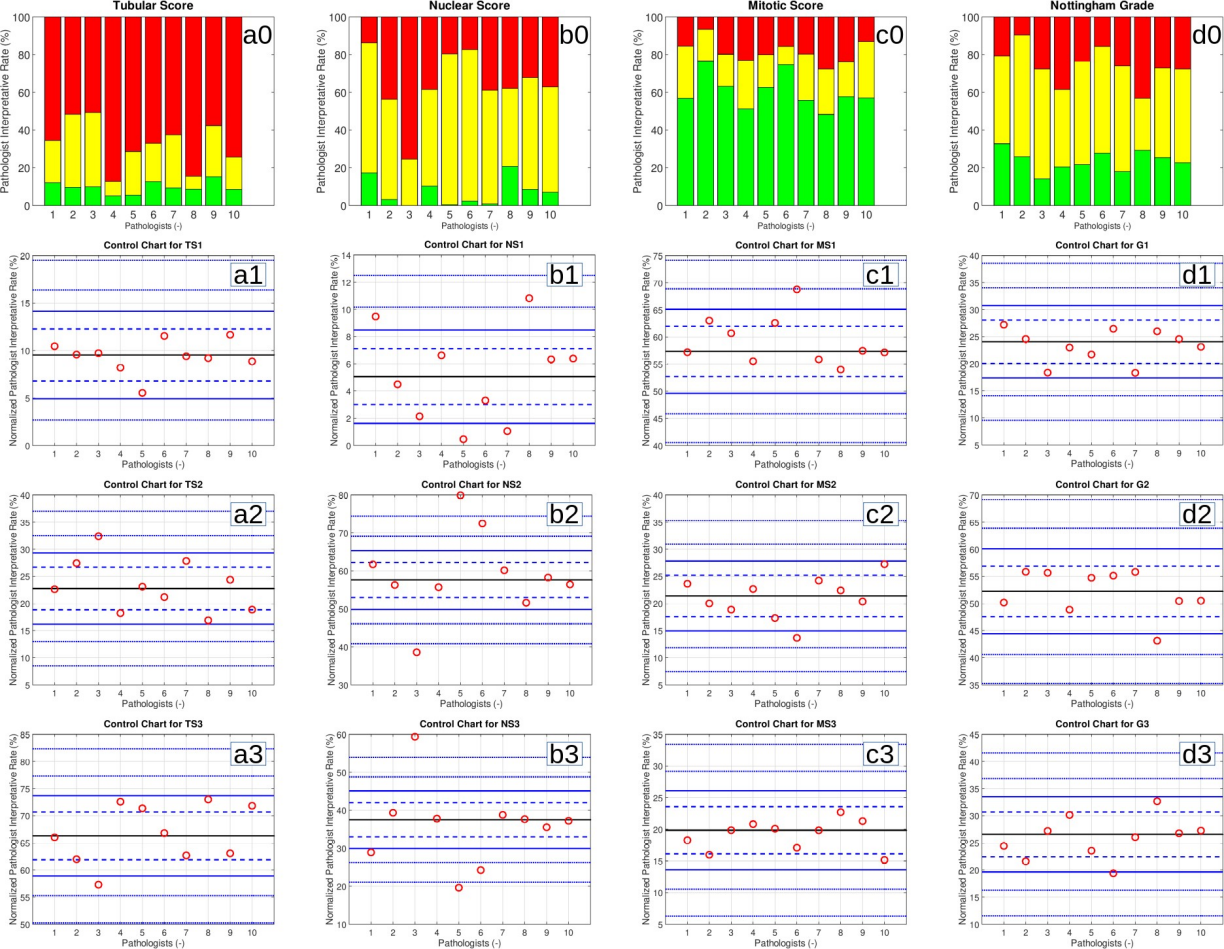
The top row (a0-d0) shows the raw PIRs in bar charts for the components of the Nottingham grade (tubular score (TS), nuclear score (NS), mitotic score (MS)) and the Nottingham grade. Green is score 1, yellow is score 2 and red is score 3. The control charts (a1-d3) show the normed PIRs for the components of the Nottingham grade (TSx, NSx, MSx) and the Nottingham grade (Gx). The bars (in the bar chart) and red circles (in the control charts) represent individual pathologists which are ordered randomly. Pathologists in the bar charts (e.g. pathologist #1) correspond to pathologists in the control charts. The black line in the centre of the control chart is the group median interpretative rate (GMIR). The dashed blue (control) lines on either side of the GMIR represent the boundaries of the 95% confidence interval (CI) and correspond to p = 0.05. The solid blue (control) lines represent the boundaries of the 99.9% CI and correspond to p = 0.001. The dashed blue (control) lines beyond the solid blue lines represent p = 1e-6 and p = 1e-12 respectively.

The PIRs were visualized with funnel plots (not shown) and control charts (see Fig 1). It should be noted that the bar charts have the (raw) PIR. The control charts have the normalized PIR. For each data point, the normalized PIR is the same number of standard deviations from the GMIR as the (raw) PIR.

### Limitations

The utility of this approach is limited by the case volume. The pathologist interpretative rates (PIRs) of low volume pathologists is not well-estimated; thus, this technique is best suited to high volume specimens in high volume practices.

The detection of differences is essentially limited to the width of the funnel in the funnel plots.

The best pathological interpretation rate/target rate cannot be determined from the pathology reports alone and is unknown.

During the time period analyzed, no ancillary tests (e.g. Ki-67 immunostaining) were used by any of the pathologists routinely as an adjunct to the histomorphological assessment of the grade. Thus, the possible effect of ancillary tests on the grading consistency cannot be assessed.

Characteristics of the pathologists were not part of the ethics approval; thus, this information was not available for analysis. Pathologist experience, and training may be significant predictors; however, this analysis did not consider these factors. This may be explored in future work.

## Discussion

The extraction procedure is largely automated. Thus, as the computer code has been written, it was modest work to analyze this data set.

### Comparison to case reviews

This approach scales well with volume vis-à-vis case reviews; a higher case volume does not require more resources proportional to the increased volume and has more statistical power to detect differences.

Case reviews may be confounded by bias; possible confounders are: case selection bias, follow-up bias, reviewer interpretation bias, confirmation bias (if the reviewer knows the primary pathologist’s interpretation), confirmation bias of a secondary reviewer (if they are not blinded to the fact that they are doing a tertiary review where the initial review yielded a discordant interpretation).

The approach herein cannot identify interpretative differences for an individual case; however, it does provide a robust framework for detecting systematic interpretative differences that are clinically relevant but may be relatively infrequent, e.g. occur in 10–15% of cases. Random quality reviews of 1–2% are statistically under-powered to reliably detect differences of this size.

Observational data does not replace case reviews; however, it can be used to target them, and can identify issues that may be expensive to detect via unselected case reviews.

### ISK and conventional kappa studies

The ISK findings partially mirror trends of conventional kappa studies (CKS) in the literature (e.g. ISK for TS > ISK for NS). The relatively poor CKS scores in the literature for MS (vis-à-vis TS and NS) is different than seen with (OMECA) ISK for MS. We suspect that this may be due to case selection. In unselected breast cancer cases MS1 is predominant; study sets for CKS (that are selected to have more MS2 and MS3 cases) may have more cases that sit at the mitotic score boundaries and generate more disagreements than actually seen in practice.

The intermediate score in a 3-tier system is often expected to have the most variability. TS is the only component of the grade that follows this pattern. The nuclear score has the most outliers; this may be explained partially by the fact that NS1 is inconsistently used.

Overall, the pathologist interpretative rates for the Nottingham grade (G) are very similar (OMECA ISK = 0.71) and few statistical outliers were present. Seen from a design perspective, a predictor (like Nottingham grade) with multiple components is likely more robust/reliable than a one component predictor. In this context, it is not surprising that the OMECA ISK for the grade is higher than that of the components (TS, NS, MS).

Direct comparisons between CKS and ISK should be done with caution, especially if the study populations and pathologists are different. Also, OMECA and MCOA should be understood as best case scenarios for a given set of interpretative rates; thus, an ISK would be expected to be higher than a CKS, if compared head-to-head in the same patient population with the same pathologists. It should be noted that a CKS using this set of cases and the ten pathologists would be impractical. It would require the ten pathologists to each examine 1,636 complete breast cancer resections; this would be 16,360 interpretations!

The advantage of ISK is: the cases are unselected and the analysis is based on the assessment of whole cases. ISK may be an intuitive metric for pathologists, as the conventional kappa is understood (at a conceptual level) by many pathologists; also, kappas are relatively common in the pathology literature.

### Control charts

The control chart analysis herein is visual (Fig 1). As pathologists spend a significant amount of time interpreting images, the analysis may be accessible without much effort. A more numbers focused assessment of the data would make use of *ordinal logistic regression*. This was done using R (https://cran.r-project.org/); it yields similar trends (analysis not shown).

### Predicting outcomes and quality

Pathologic classifications are valuable as they predict outcomes. Their value is derived from how other patients were classified and what their outcome was. Variations in the interpretation/classification reduce the predictive power if one examines the issue at the cohort level. At the level of the patient, variations in interpretation lead to non-optimal treatment that is not appropriately tailored to the disease severity. Measuring PIR and comparing to internal and external benchmarks, is the basis for an informed discussion about diagnostic consistency.

Oncologists use the Nottingham grade when determining which patients could potentially benefit from adjuvant systemic chemotherapy [18]. As systemic chemotherapy for breast cancer is associated with significant side effects, it is important to achieve high grading accuracy, as it avoids unnecessary toxicities [18]. Retrospective analyses have been performed comparing primary pathologist grade and a central review grade [18,19]. Substantial inter and intra-laboratory variation was identified in histologic grading [18].

Given the variation in grading that is seen in the literature and in our own environment, consideration of a central review of grading may be justified to reach the best possible histologic grade. However, the resources to do central reviews are not insubstantial.

A significant advantage of the approach herein is: pathologists could get insight into their interpretative rates without having to go through a larger study set and could compare themselves to (1) their colleagues and (2) values in the medical literature.

The variation in grade appears to have several underlying causes, that are dependent on the individual pathologist. There does not appear to be a one-size-fits all piece of advice that can be given to all pathologists. Pathologists often do not know their own interpretative rates and rarely know how they compare to their colleagues or other institutions. It is an old cliché: “[i]f you don’t measure you don’t know”. As data analyses of this type become more widespread, interpretative rate will likely become more important. Interpretative rates are a rational starting point for assessing diagnostic variance and “calibrating” or optimizing future interpretations.

What the individual pathologist may or may not have to do to improve depends on: (1) how they practise, and (2) what the best call is for the population they serve–which may be inferred from how they measure-up against their colleagues, local (breast pathology) experts and literature values.

Future work will include (1) providing individualized feedback to the pathologists, (2) reviewing grading criteria and (3) tracking the grading parameters—with the goal of increasing the consistency of the grade and the individual components of the grade; in short: applying statistical process control/Next Generation Quality to the Nottingham grading.

The Nottingham grade when combined with the tumour receptor status (e.g. progesterone receptor) and other pathologic parameters (e.g. tumour size) has been shown to predict molecular testing, such as “OncotypeDx” [20–22]; thus, if grading is done with a higher level of reproducibility within a controlled process, it can corroborate other (cost-intensive) tests and may allow more effective resource use with a higher level of quality.

## Conclusion

The individual pathologist is a predictor of Nottingham grade; this suggests that consistency could be improved. If pathologists are aware of their interpretative rates, a statistical process control framework/Next Generation Quality could improve grading and improve the prognostic value and reliability of the Nottingham grade.

## Supporting information

S1 File (PDF)

S1 Data (CSV)
