# Supplementary material for: Variation in breast cancer grading in 1,636 resections assessed using control charts and in silico kappa
Source: PLoS One. 2020 Dec 28;15(12):e0242656. doi: 10.1371/journal.pone.0242656 (PMC7769472; doi:10.1371/journal.pone.0242656)
Supplement: S1 File — (PDF) [file pone.0242656.s001.pdf]

Visual Explanations of the Funnel Plot Normalization, MCOA and OMECA

Funnel Plot Normalization

Normalization – objectives:
1. Simplify plots (remove case volume)
2. Maintain the number of standard deviations to the group median interpretative rate (GMIR)

The normalization procedure amounts to moving a point along a curve that defines a constant number of standard deviations (SD) to the GMIR (green dashed curve):

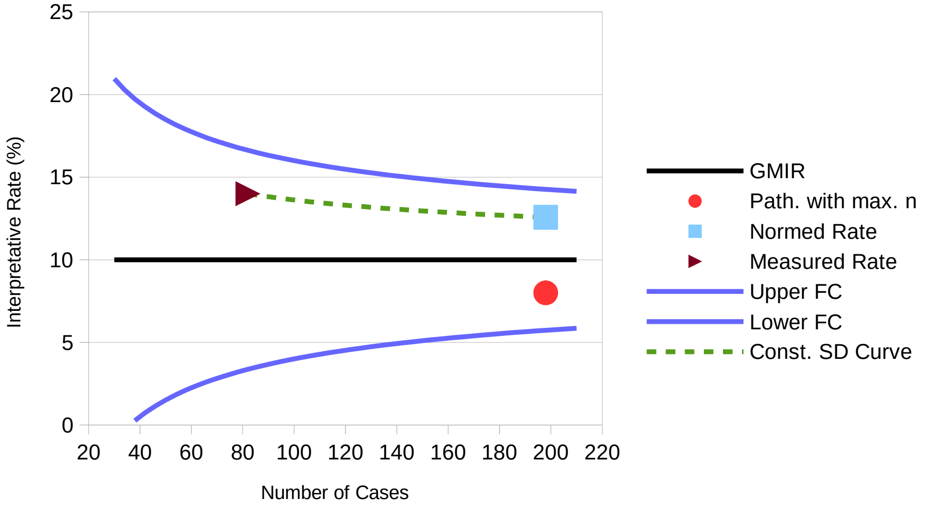

Figure 1: Normalization of Measured Rate (82,14). 'FC' is funnel curve. 'SD' is standard deviation. The red dot represents the pathologist that interpreted the most cases.

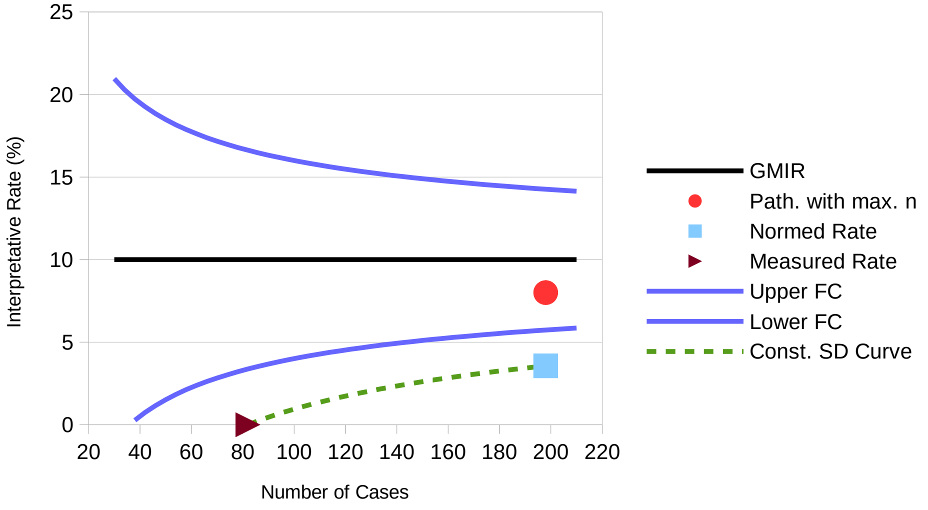

Figure 2: Normalization of Measured Rate (82,0). 'FC' is funnel curve. 'SD' is standard deviation. The red dot represents the pathologist that interpreted the most cases.

Maximal Category Overlap Assumption (MCOA)

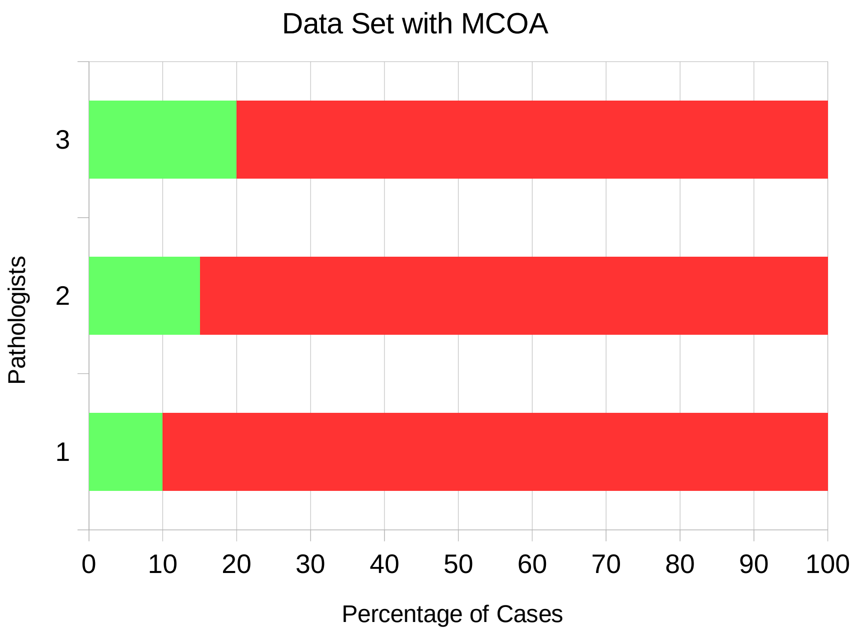

Figure 3: Data set with MCOA. All pathologists with a higher call rate call the cases that a lower call rate pathologist calls.

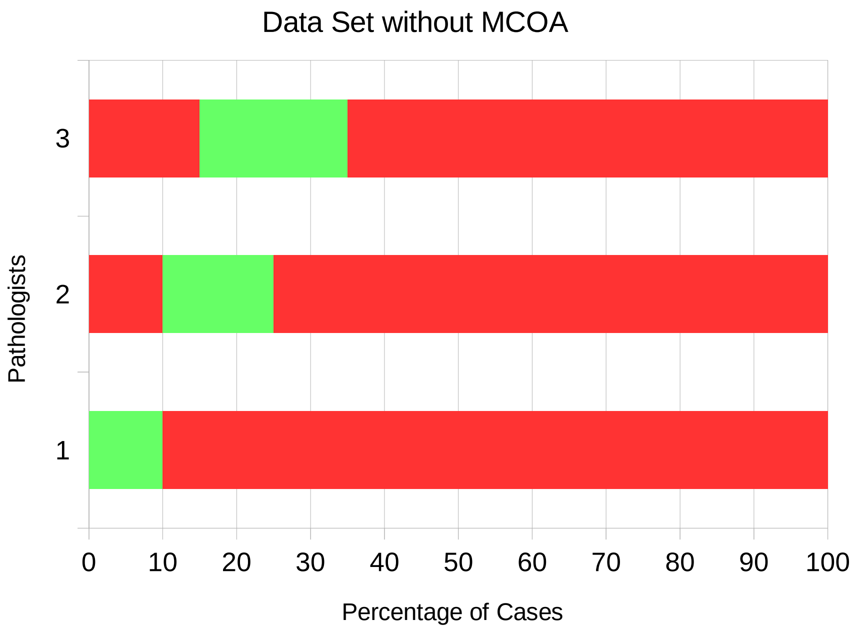

Figure 4: Data set without MCOA. Pathologist #2 and #3 have partial overlap. Pathologist #1 has no overlap with the others.

Ordered Mutually Exclusive Category Assumption (OMECA)

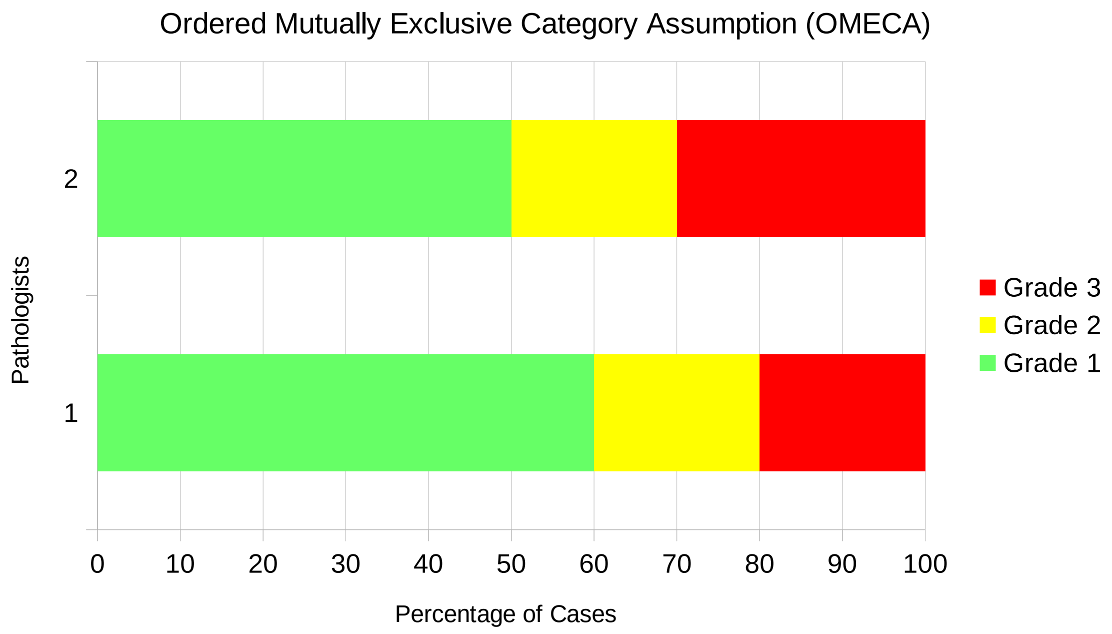

Figure 5: Data set with ordered mutually exclusive categories.

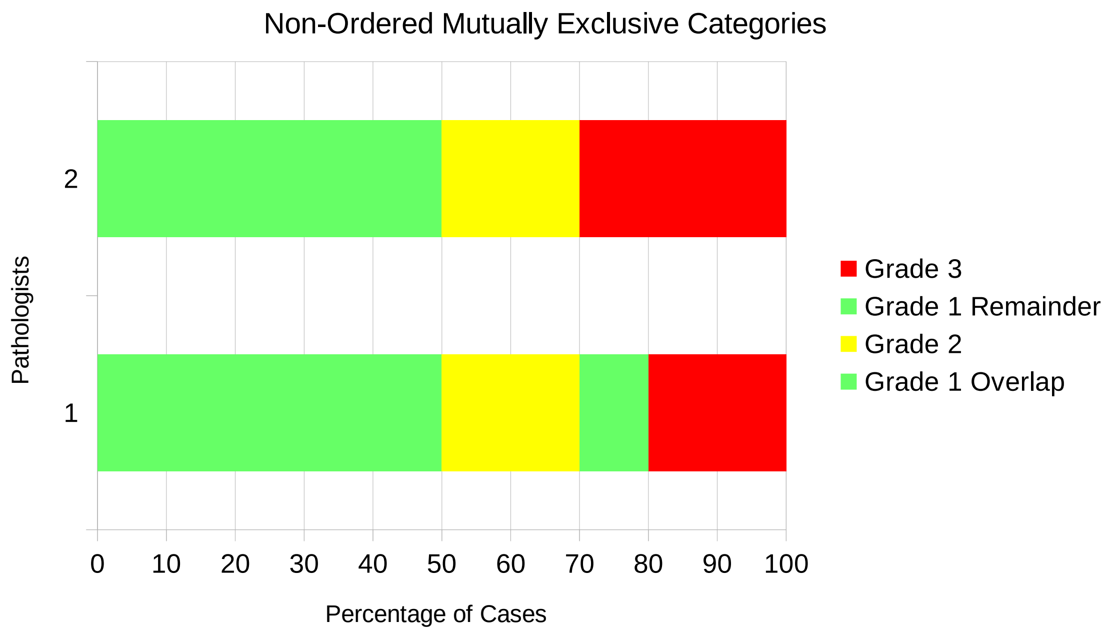

Figure 6: Data set where mutually exclusive categories are not ordered. As the categories are ordered the above arrangement would be unlikely, as one usually chooses between adjacent categories (1 grade vs grade 2 or grade 2 vs grade 3, not grade 1 vs grade 3). (Note: This data shows maximal categorical overlap.)
